# Development of a Novel Low-Calorie Lime Juice-Based Prebiotic Beverage Using a Combined Design Optimization Methodology

**DOI:** 10.3390/foods12030680

**Published:** 2023-02-03

**Authors:** Leila Abolghasemi Fakhri, Babak Ghanbarzadeh, Pasquale M. Falcone

**Affiliations:** 1 Department of Food Science and Technology, Faculty of Agriculture, University of Tabriz, Tabriz P.O. Box 51666-16471, Iran; 2 Food and Environmental Sciences, Department of Agricultural, University Polytechnical of Marche, Brecce Bianche 10, 60131 Ancona, Italy

**Keywords:** high-fiber prebiotic beverage, lutein, lime peel essential oil, peppermint extract, optimization

## Abstract

A novel lime-juice based low-calorie functional beverage was developed by using D-optimal combined design optimization. For the preparation of the beverage, the following functional ingredients were used: lime juice, lime peel essential oil (LEO) as a flavoring agent and bioactive component, sucralose as a low-calorie sweetener, an inulin/polydextrose (I/P) mixture as prebiotic fibers, pectin as a thickening agent and soluble dietary fiber, lutein as a carotenoid colorant and antioxidant, and peppermint extract (ME) as a flavoring agent and bioactive component. A combined design consisting of one mixture factor (LEO/ME ratio), one numeric factor (lutein concentration), and one categoric factor (presence or absence of prebiotics) was used for optimizing the functional beverage based on the sensory quality. Regression models were adequately fitted to the data of sensory acceptance with a determination coefficient >90%. The sample containing a mixture of prebiotics, 2:3 (*v/v*) ratio of LEO: ME, and 3 mg/100 mL lutein was selected as the best formulation among the six optimal beverages which was suggested by Design-Expert software. This final optimum sample showed the highest total phenolic (44.22 mg gallic acid equivalents/L) and flavonoid (25.49 mg quercetin equivalents/L) contents, and its antioxidant activity (as 2,2-diphenyl-1-picrylhydrazyl radical (DPPH^•^) scavenging) was 38.30%. The newly designed beverage has the potential to promote health benefits and in therapeutic applications.

## 1. Introduction

Functional beverages such as dairy drinks, energy drinks, sports drinks, ion drinks, herbal teas, and fruit- and vegetable-based beverages are non-alcoholic drinks that are enriched with nutraceuticals, in addition to the basic nutritional value of the product, to provide multiple health-related benefits. These functional ingredients include amino acids, vitamins, minerals, antioxidants, essential fatty acids, phytonutrients (such as carotenoids, polyphenols, terpenes, and phytosterols), fibers, prebiotics, and probiotics. Consumer interest in functional foodstuffs that are not only highly nutritious and healthy but also easy to prepare and ingest has led to the growing popularity of fruit-based functional beverages. Unfortunately, most conventional functional beverages, such as energy drinks, sports drinks, and fruit beverages, are sweetened and contain significant amounts of sugar and hence, consuming them can potentially increase the risk of diabetes, obesity, and heart disease. Using non-nutritive-high intensity sweeteners such as sucralose can meet consumer demand for no/low-sugar content products aimed at obesity prevention, weight control, and diabetes management [1,2]. However, some properties of sucralose-sweetened beverages, including taste, aftertaste, bulking, flavor, mouthfeel, and structure, are still significantly different from regular beverages. Among the artificial sweeteners approved by the European Union (EU) for use as food additives, aspartame, acesulfame, sucralose, and saccharine are the most widely used. Sucralose has recently overtaken traditional saccharine and aspartame as the most widely used artificial sweetener due to its sugar-like sweetness, high sweetness intensity (about 600 times that of sucrose), stability, non-nutrition, and safety (the majority of ingested sucralose is not metabolized and absorbed by the human body) [3,4,5]. A recent study has highlighted sucralose as a valuable alternative to sucrose and has shown that sucralose contributes to the prevention of a diversity of metabolic disorders and health constraints [6]. In general, it is necessary to control the amount of artificial sweeteners for consumer safety [3].

Lime (*Citrus aurantifolia*) is a good source of nutrients and bioactive compounds which can be useful for the positive regulation of oxidative stress, lipid profiles, and inflammatory cascades. Moreover, due to its high flavonoids content, lime juice can potentially exert neuroprotective effects and become general dietary brain food [7,8,9]. Lime juice is a rich source of vitamin C and citric acid and has a low pH, and in contrast with other types of citrus fruits, such as grapefruits, tangerines, and oranges, it has a higher percentage of citric acid than sugar content, all of which give it a highly acidic flavor and sour taste [10,11]. The highly acidic lime juice with a distinct aroma and flavor and a unique sour taste, is often used to enhance the flavor and aroma of foods and beverages. Therefore, it can be a suitable matrix for developing new value-added beverages.

Inulin and polydextrose as soluble prebiotic fibers play an important role in promoting health and preventing diseases, including improving digestive health and function, inhibiting the proliferation of harmful microorganisms and improving the growth and activity of beneficial intestinal bacteria, attenuation of postprandial blood glycemic and insulinemic response, reducing calorie intake, and the risk of obesity and type 2 diabetes and related disorders [12,13]. These prebiotics can be used in transparent, low glycemic, and sugar-free beverages suitable for people with diabetes [14]. Peppermint (*Mentha piperita* L., from Lamiaceae) extract is one of the most important medicinal and aromatic herbal extracts with distinguished bioactive potential. It is a blood sugar regulator and has shown significant inhibition against key enzymes of type 2 diabetes (α-glucosidase) and hypertension (angiotensin 1-converting enzyme, ACE) [15]. Lime (*C. aurantifolia*) peel essential oil (LEO) is a commercially important citrus essential oil (EO) (the most important by-product of citrus processing) with high nutraceutical, antioxidant, and sensory characteristics, as well as economic importance. The findings suggest that LEO can affect food intake and a diverse array of processes involved in energy expenditure and fuel utilization, all of which suppress weight gain [16]. LEO is classified as Generally Recognized as Safe (GRAS) according to the Food Additive Status List [17] and is a valuable product for flavoring purposes. Lutein, a natural bioactive colorant and potent antioxidant, is known to play an established role in eye health and a protective role against cardiovascular and chronic diseases, cancer, etc. [18,19]. Positive effects of lutein on health issues such as age-related macular degeneration (AMD) have been reported at dietary intake levels of 6–14 mg/day [20].

Sensory acceptability is a crucial factor when designing newly enriched foodstuffs, and customer acceptance issues are required to be overcome at first. Combined design (CD) is a versatile experimental design technique that can be applied to operate multi-objective optimization design under reduced experimental runs. It has recently been used as one of the most popular methods for optimizing product formulation in the food industry [21]. Recently, studies have been conducted on the formulation and evaluation of the properties of new functional fruit-based beverages and sugar-free products [22,23,24,25,26]. The chemical compounds of cold-pressed LEO and their antioxidant effects as well as their contribution to sensory properties have been investigated [9,27]. The development of a drink that is low in calories, high in fiber, and rich in antioxidant bioactive compounds, and also has good sensory characteristics seems to be very interesting from a health aspect. The objectives of the current research were to develop a low-calorie functional beverage enriched with inulin/polydextrose prebiotics, lutein, LEO, and ME with high consumer acceptance. For this purpose, the effects of functional ingredients and their level of use on sensory quality were modeled, and the formulation of the beverages was optimized using the CD approach. Finally, the beverage with the highest bioactive compound content in terms of the total phenolic and flavonoid contents and antioxidant potential was selected as the final optimum formulation.

## 2. Materials and Methods

### 2.1. Materials

Lime (*C. aurantifolia*) concentrate (∼45° Brix), lime (*C. aurantifolia*) peel essential oil (cold-pressed), and peppermint (*Mentha piperita* L.) extract was provided from Takdaneh Co. (Tabriz, Iran), all stored in a dark container at 4 °C until use. Inulin (from chicory roots, 92.52% pure, long-chain inulin (≥ 23 monomers)) and polydextrose (95.5% pure) were obtained from Pyson Co., Ltd. (Shaanxi, China). High-methoxyl (HM) pectin (galacturonic acid, ≥74.0% on a dry basis) and all other chemicals were supplied from Sigma-Aldrich (Germany), and all were of analytical grade.

### 2.2. Preparation of the Beverages

The protocol for the preparation of 1 L of beverages in a laboratory scale is shown in Figure S1. The final product contains 10% *w*/*v* of “reconstituted lime juice with 8.3 °Brix”. The concentration of sucralose was equivalent in sweetness to 10% *w*/*v* sucrose based on a previous study [28]. LEO, ME, and lutein were added at concentration ranges determined based on sensory analysis, and depending on the CD points (Table 1). A beverage must contain 6 g or more of dietary fiber per serving (20% of the daily reference value (DRV)) to be considered “high in fiber” [29]. The DRV for fiber is 30 g per day based on a 2500-calorie diet. Considering the purity of inulin and polydextrose, beverages were formulated with 2.85 and 2.78% *w*/*v* inulin and polydextrose, respectively, to meet the recommendation of providing 6 g inulin and polydextrose fibers in serving sizes of 240 mL [29]. All sample preparations were carried out in triplicate.

### 2.3. Determination of Physicochemical Properties

#### 2.3.1. Extraction of Phytochemicals

The antioxidant capacity, total flavonoid content (TFC), and total phenolic content (TPC) were assessed on an extract of antioxidants from beverage specimens, all as described previously [30,31]. Two milliliters of each sample was homogenized with 10 mL ethanol (80% *v/v*) using a magnetic stirrer (SH-2, Topline Lab). The homogenate was centrifuged at 13,500 g for 15 min at 4 °C using a CR22N centrifuge (Hitachi Koki Co., Ltd., Tokyo, Japan). The supernatant was collected and filtered through a Whatman #1 filter paper. The ethanolic extract was stored at −20 °C for analysis. All assays were performed in triplicate.

#### 2.3.2. Total Phenolic Content (TPC)

A 200 μL sample of extract to water (1:20) was added to 1 mL of the Folin–Ciocalteu reagent (FCR) (diluted 1:10). After 3 min of incubation at 20 °C, 800 µL of 7.5% Na_2_CO_3_ solution was added followed by the incubation of reaction mixture at the same temperature for 2 h. The absorbance at 765 nm was determined by a UV–Vis double-beam spectrophotometer (Shimadzu UV-1700, Kyoto, Japan), and the TPC was calculated using gallic acid as the standard. The calibration curve of the gallic acid was created at 10–200 mg/L, and the TPC was reported as mg gallic acid equivalents (GAE)/L of the specimen.

#### 2.3.3. Total Flavonoid Content (TFC)

In brief, the ethanolic extract (0.2 mL) was mixed with deionized H_2_O (1.28 mL) and NaNO_2_ (0.06 mL, 5%). After 5 min at 20 °C, AlCl_3_ (60 μL, 100 g/L) was incorporated, and 6 min later, NaOH (0.4 mL, 40 g/L) was added under the same conditions. The mixtures were stirred and the absorbance was measured at 510 nm using a UV–Vis double-beam spectrophotometer (Shimadzu UV-1700, Japan). The TFC was calculated based on the calibration curve of quercetin (10–200 mg/L), and the results were expressed as mg quercetin equivalents (QE)/L of the sample.

#### 2.3.4. Antioxidant Capacity by the DPPH^•^ Scavenging Assay

A 100 µL sample of ethanol was mixed with 3.9 mL of ethanolic DPPH^•^ solution (39.43 mg/L) (blank) to determine the initial absorbance of the DPPH^•^ solution. Then, 100 µL of ethanolic extract was added to 3.9 mL DPPH^•^ ethanolic solution (39.43 mg/L). The mixture was shaken immediately and incubated at 20 °C in the dark. After one hour, the decrease in absorbance at 517 nm was measured using a UV–Vis double-beam spectrophotometer (Shimadzu UV-1700, Japan). The DPPH^•^ scavenging activity was expressed as the inhibition percentage of the DPPH^•^ using the following equation:

(1) $$Radical\;scavenging\;activity\;\left(\%\right)=\frac{A_{0}-A_{s}}{A_{0}}\times 100$$

where *A*_0_ and *A_s_* correspond to the absorbance of the control blank and sample, respectively.

#### 2.3.5. Ascorbic Acid Content (AA)

The ascorbic acid content of samples was quantified using the iodine titration method as described by taking 0.88 mg AA, equivalent to 1 mL of iodine solution (preparation details are given in the Supplementary Material) [32]. Then, 20 mL of the samples were mixed with 150 mL of distilled water and titrated with iodine solution in the presence of 1% starch solution as an indicator until the solutions reached a fixed dark-blue color. All measurements were run in triplicate. AA content was estimated using Equation (2):

(2) mg ascorbic acid/100 mg sample=0.88×ml iodine solution

#### 2.3.6. Total Soluble Solids (TSS), pH, and Titratable Acidity (TA)

The pH and TSS of samples were measured at 20 °C using a refractometer (Mettler Toledo, Greifensee, Switzerland) and a pH meter (Mettler Toledo, Greifensee, Switzerland), respectively. The TA determination details are given in the Supplementary Material. All tests were performed in triplicate.

### 2.4. Sensory Analysis of Beverage Samples

Sensory evaluation was performed by a panel of thirty semi-trained members using a 9-point hedonic scale ranging from 1 (dislike extremely) to 9 (like extremely) [26]. These panelists (aged 23–40 years) were students of the Department of Food Science and Technology, University of Tabriz, Iran. Beverage samples (40 mL) were served at 4 °C in transparent polyethylene cups, coded with 3-digit random numbers. The samples were arranged in a randomized order and the panelists were asked to evaluate and score for taste, flavor, texture, color, and overall acceptance attributes. Potable water and salt-free crackers were provided as palate-cleansing agents. The reported values were the average of the three analyses.

### 2.5. Experimental Design and Statistical Analysis

A D-optimal combined design having two mixture components (LEO (diluted 1:10) and ME concentrations), one numeric factor (lutein concentration), and one categoric factor (I/P mixture at two levels, absence (level 1) or presence (level 2) of fibers) was applied, and 17 experimental points were obtained (Table 1). The data were analyzed and the contour and 3D surface plots were created by Design-Expert package software (Version 10, Stat-Ease Inc., Minneapolis, MN, USA). The following equation was fitted to the data:

(3) $$Y=\beta_{0}+\sum_{i=1}^{k}\beta_{i}X_{i}+\sum_{i=1}^{k}\beta_{ii}X_{i}^{2}+\sum_{\begin{array}{c} i=1\\ i<j \end{array}}^{k-1}\sum_{j=2}^{k}\beta_{ij}X_{i}X_{j}$$

where *Y* is a response variable, *k* is the number of variables, *X_i_* and *X_j_* are independent variables in coded units, *β_ij_*, *β_ii_*, and *β_i_* are the measures of the *X_i_*, *X_j_*, $X_{i}^{2}$, and *X_i_X_j_* of linear, quadratic, and interaction effects, respectively, and *β*_0_ is a constant coefficient.

The TPC, TFC, and antioxidant data were analyzed through analysis of variance (ANOVA) using SPSS software (Version 22, SPSS Inc., Chicago, IL, USA), and significant differences (*p* ≤ 0.05) were identified by Duncan′s multiple range tests.

### 2.6. Overall Optimization of the Variables

The graphical and numerical optimization methods were carried out using Design-Expert 10 software based on the desirability function approach for maximum sensory acceptance.

### 2.7. Verification Experiments and Validation of the Model Equations

Verification tests (three replicates) using the optimal amounts of independent variables were used to confirm the adequacy of the equations obtained. The last optimal beverage formulation was developed using the same process mentioned above (Section 2.2). The difference between the optimum point of the beverage sample and its repeat in terms of the studied sensory characteristics was evaluated by sensory analysis using the same group of panelists (Section 2.4). To assess the validity of the regression models, the actual data were compared with the values predicted by the models.

## 3. Results and Discussion

### 3.1. Optimization of the Sensory Properties of the Beverages

#### 3.1.1. Analysis of Regression Models

Table 1 presents the experimental design, independent variables levels, and the experimental and predicted values for the responses. Data indicated that all the sensory properties are generally affected by the formulation of the beverages. Various mixture–process combined models were fitted to the experimental data to obtain the regression equations. The sequential p-value, model summary statistic (MSS), and lack of fit tests (LOF) were used to evaluate the model adequacy (Table 2). Based on the significant sequential p-value (*p* < 0.01), insignificant LOF (*p* > 0.05), and the highest determination coefficient (R^2^), adjusted R^2^ (adj-R^2^), and predicted R^2^ (pre-R^2^) amongst the models tested, the quadratic × linear model was chosen as the most appropriate model for taste, color, and overall acceptance. In contrast, the quadratic × mean and linear × linear models were selected for flavor and texture, respectively (Table 2).

The quality of fit and adequacy of developed models was then checked and verified by ANOVA and regression analysis (Table 3). The highly significant model (*p* ≤ 0.001) and non-significant lack of fit (*p* > 0.05) showed the adequacy of models developed for all responses used to constitute the correlation between variables and responses. High R^2^ values (>80%) suggest that all the models have a good fit and could describe the effect of variables on the responses. Reasonable agreement between the pre-R^2^ and the adj-R^2^ for all responses indicated the adequate accuracy and general availability of the models. The coefficient of variation (C.V.) was less than 10% for all responses which provided better reproducibility and indicated a high degree of precision and a good deal of reliability of the experimental values. The adequate precision values (measure of the signal/noise ratio) greater than 4 for all responses indicate adequate signals [33]. The results showed that the models made in this study are reasonable for analyzing the responses.

The linear and interaction effects of *X*_1_ and *X*_2_ were the significant parameters associated with taste, flavor, and color models (*p* < 0.01) (Table 3). The interaction term of *X*_1_ and *X*_4_ also has a significant effect on taste. For texture, in addition to *X*_1 × 4_, *X*_2_*X*_4_ was also a significant model term (*p* < 0.01). The interaction term of *X*_1_ and *X*_3_ also showed a significant influence on the color properties. *X*_1_*X*_2_, *X*_2_*X*_3_, and *X*_2_*X*_4_ were significant parameters for the overall acceptance model. Finally, the results indicated that the interaction effect of mixture components (*X*_1_*X*_2_) was the most significant factor affecting taste, flavor, color, and overall acceptance, and the interaction effect of LEO (*X*_1_) and prebiotic fibers (*X*_4_) was the most significant parameter affecting the texture. When comparing mixture components coefficients, LEO was found to be a more influential factor in flavor and color (Table 3 and Equations (6) and (8). Only the interaction effect of the LEO and prebiotics showed a negative effect on texture (*p* < 0.01) (Equation (7)). The final fitted equations in terms of L_Pseudo components, coded process factor, and coded categoric factor with the significance coefficients were

(4) $$Y_{1}=5.99\;X_{1}+5.42\;X_{2}+6.46\;X_{1}X_{2}+0.17\;X_{1}X_{4}$$

(5) $$Y_{2}=6.80\;X_{1}+6.29\;X_{2}+5.28\;X_{1}X_{2}$$

(6) $$Y_{3}=-1.01\;X_{1}X_{4}+0.53\;X_{2}X_{4}$$

(7) $$Y_{4}=6.35\;X_{1}+4.39\;X_{2}+3.59\;X_{1}X_{2}+0.65\;X_{1}X_{3}$$

(8) $$Y_{5}=6.58\;X_{1}X_{2}+0.49\;X_{2}X_{3}+0.40\;X_{2}X_{4}$$

where *Y_1_*, *Y_2_*, *Y_3,_ Y_4_*, and *Y_5_* are taste, flavor, texture, color, and overall acceptance, respectively.

#### 3.1.2. Analysis of the Response Surface

##### The Interaction Effects of Functional Ingredients on Taste

**Figure 1:**
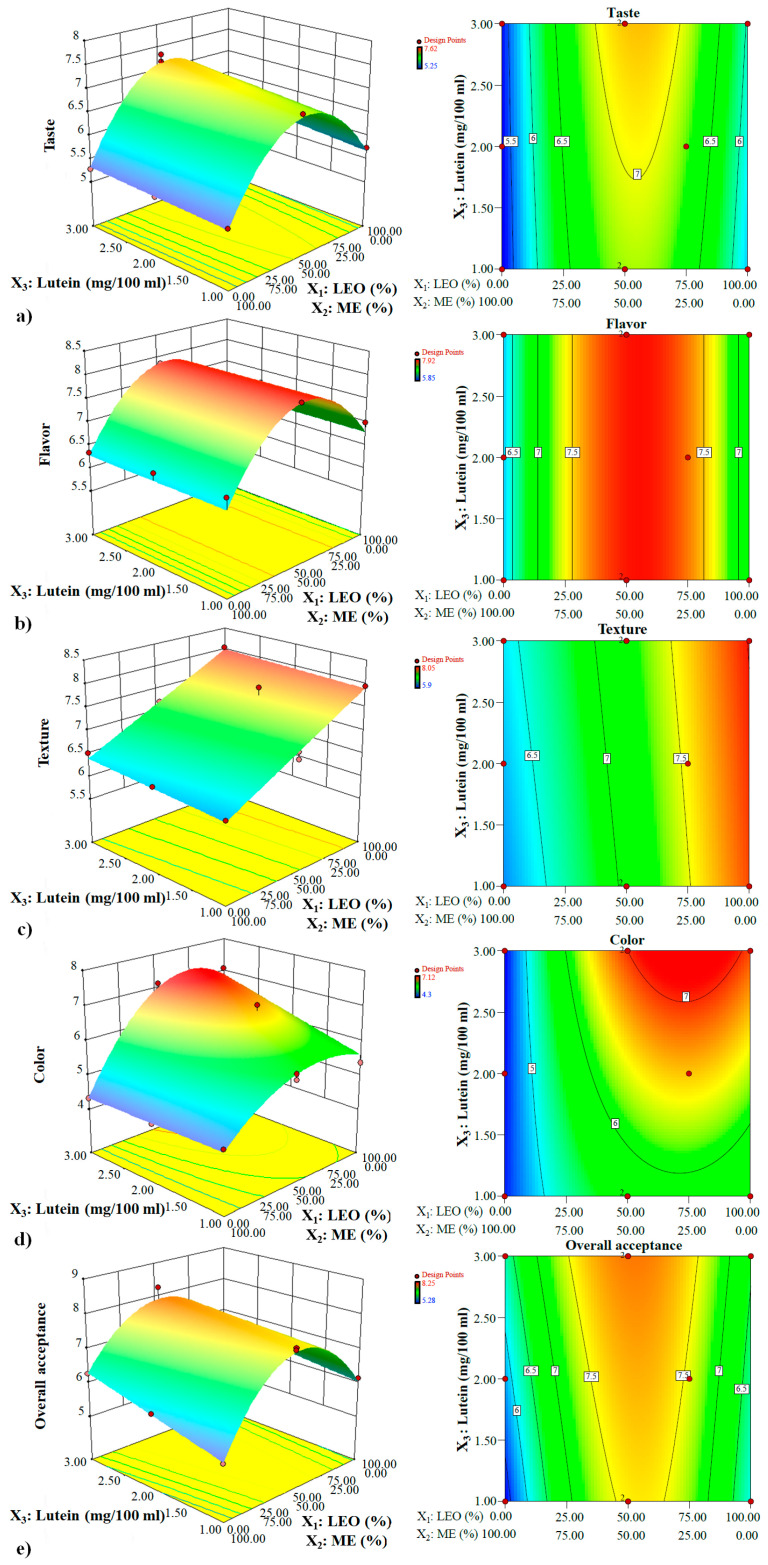
The change in (**a**) taste, (**b**) flavor, (**c**) texture, (**d**) color, and (**e**) overall acceptance based on different LEO and ME combinations under different lutein levels and in the absence of prebiotic fibers (in color).

**Figure 2:**
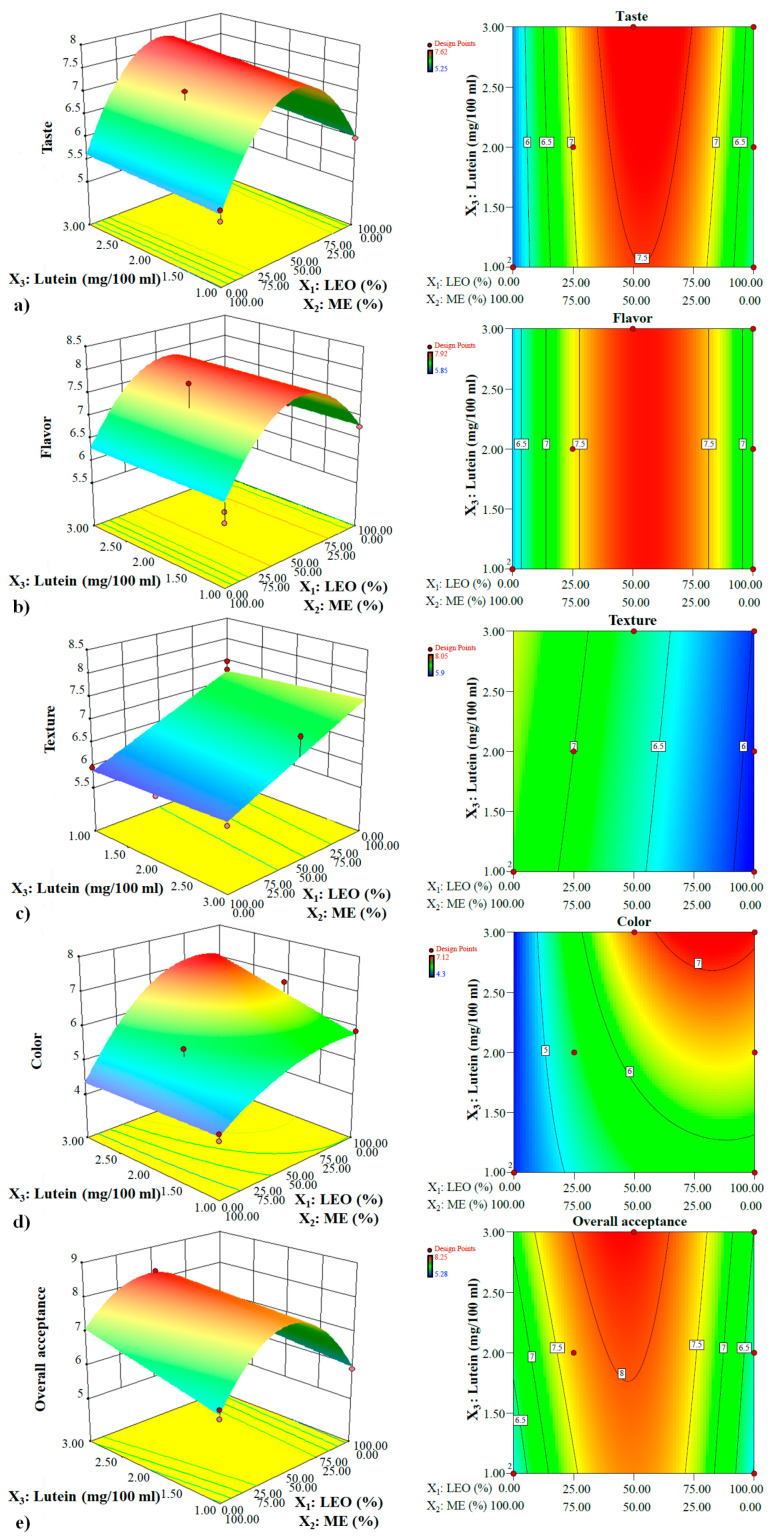
The change in (**a**) taste, (**b**) flavor, (**c**) texture, (**d**) color, and (**e**) overall acceptance based on different LEO and ME combinations under different lutein levels and in the presence of prebiotic fibers (in color).

**Figure 3:**
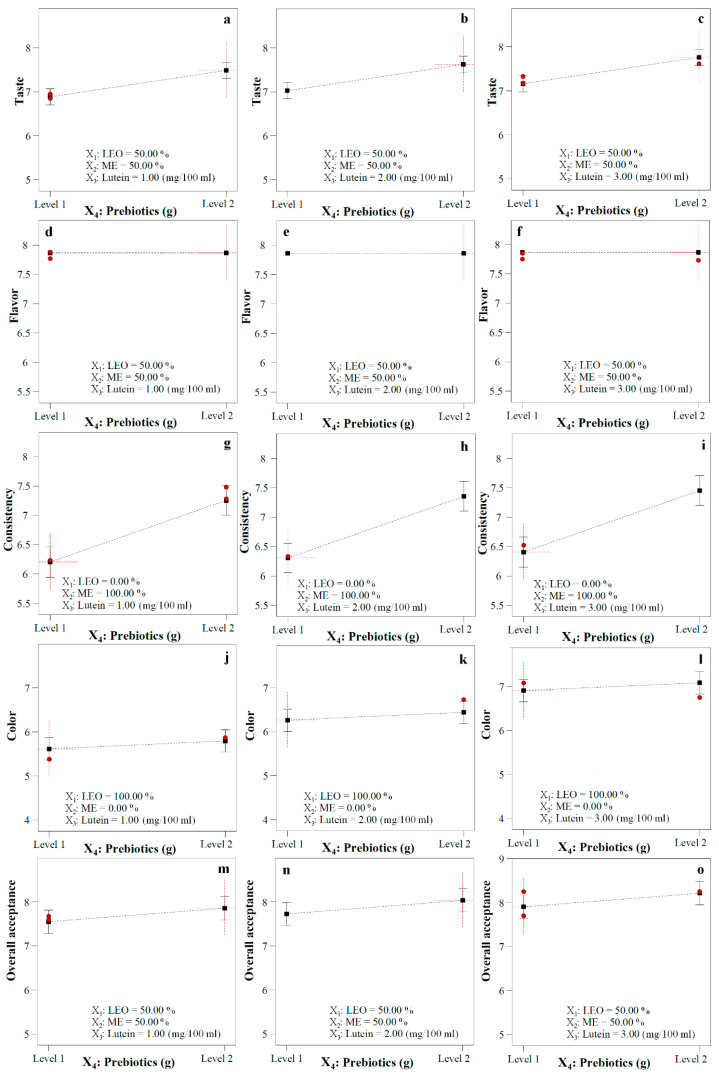
The effect of the presence and absence of prebiotics on the (**a**–**c**) taste, (**d**–**f**) flavor, (**g**–**i**) texture, (**j**–**l**) color, and (**m**–**o**) overall acceptance of beverages at specified lutein content acquired using a constant LEO/ME combination which is mentioned in the figures (in color).

A beverage containing intermediate levels of LEO and ME (approximately 55: 45 LEO: ME) showed, in general, the highest taste acceptance at all levels of lutein and in the presence or absence of fibers (*p* < 0.05) (Figure 1a and Figure 2a). The convex surface indicated a quadratic effect of LEO/ME combinations on the taste. At all amounts of lutein, taste improved with increasing LEO in the mixture up to a certain extent, but decreased with a further increase. The samples containing 100% ME in the mixture had a minimum taste acceptability score. Except at very low concentrations of LEO, lutein levels had a non-significant positive linear effect on taste acceptance of both fiber-free and fiber-enriched samples. The fibers improved taste acceptance at all lutein levels Figure 1a, Figure 2a, and Figure 3), and the LEO and fibers had a positive interaction effect on taste acceptance (*p* < 0.05) (Figure 1a and Figure 2a, and Equation (4)). When the effects of both lutein levels and fibers were considered, it was concluded that at all proportions of LEO, increasing lutein levels and adding fibers resulted in higher taste acceptance.

##### The Interaction Effects of Functional Ingredients on Flavor

At all levels of lutein, the LEO/ME combination affected the flavor in a second-order manner. Up to a critical LEO proportion in the mixture, flavor acceptance increased, while at higher ratios, a negative trend occurred (Figure 1b and Figure 2b). Flavor acceptance values behaved almost identically at all lutein levels. Neither the lutein level nor the addition of fibers caused a change in flavor acceptance (Figure 1b, Figure 2b, and Figure 3), and only the composition of the mixture components affected this parameter. At all amounts of lutein and in the presence or absence of fibers, high flavor acceptance values were obtained using 30–80% LEO in the mixture.

##### The Interaction Effects of Functional Ingredients on Texture

LEO/ME combination and lutein concentration demonstrated a linear effect on texture acceptance (Figure 1c and Figure 2c). At all concentrations of lutein and in the absence of fibers, texture showed the highest acceptability at the highest proportion of LEO in the mixture (*p* < 0.05). However, its values decreased linearly with increasing LEO in fiber-enriched beverages. The optimum ratio of LEO in the mixture was sharply reduced with adding fibers. The ME proportion and fibers had a positive interaction effect on this property (*p* < 0.05) (Figure 1c, Figure 2c, Figure 3, and Equation (6)) and at all lutein levels, fortifying with fibers increased texture acceptance at the ME concentrations higher than approximately 70% *v/v* (LEO ≤ 30% *v/v*) (Figure 3); however, at lower concentrations a rapid decrease was observed. Considering only the effect of lutein, at all combinations of LEO/ME and in both presence and absence of fibers, as the lutein increased, the response displayed a non-significant increase. Maximum texture acceptance was obtained in the samples containing no fibers and high amounts of LEO and lutein.

##### The Interaction Effects of Functional Ingredients on Color

The LEO/ME combination indicated a second-order effect on this parameter (Figure 1d and Figure 2d). Color acceptance of the fiber-free beverage increased up to a certain proportion of LEO followed by a decrease with its further increase. In the same mixture combination in fiber-free and fiber-enriched beverages, except at very low concentrations of LEO (approximately < 10%), lutein significantly improved color acceptance. LEO and lutein had positive interaction and synergistic effect on the color acceptance up to the optimal LEO/ME combination (*p* < 0.05) (Figure 1d and Figure 2d, and Equation (7)). Fortifying with fibers increased the color acceptance at high LEO proportions (Figure 1d, Figure 2d, and Figure 3). When the effects of both lutein and fibers were considered, it was concluded that an increased in lutein level and adding fibers yielded higher color acceptance only at low proportions of ME (synergistic and positive interaction effect) (Figure 1d, Figure 2d, and Figure 3). The maximum color acceptance was yielded at 2.5–3 mg/100 mL lutein using LEO concentrations >50% in the absence of fibers and >60% in the presence of fibers. The minimum acceptability score was attained at high amounts of ME.

##### The Interaction Effects of Functional Ingredients on Overall Acceptance (OA)

A strong curvature of the surfaces pointed out the high significance of the quadratic effect of the LEO/ME combination on OA (Figure 1e and Figure 2e). OA increased when LEO in the mixture was raised to a certain extent, which was the optimum mixture combination. Beyond this value, a decrease in OA with LEO proportion was observed. At the same mixture components level, the OA score of fiber-free and fiber-enriched beverages increased with increasing lutein content (*p* < 0.05). This increase was more significant in the samples containing only ME. The optimum mixture combination changed slightly depending on the lutein concentration, and with the rise of the lutein content, the optimum mixture had more ME. Unlike very low ME proportions, at the same mixture components level, fortifying with fibers at all lutein levels increased the OA (Figure 1e, Figure 2e, and Figure 3). The ME and lutein, and ME and fibers had positive interaction and thus had a synergistic effect on OA (*p* < 0.05) (Equation (8)). Considering both the impact of lutein levels and fibers, it was concluded that increasing the level of lutein and adding fibers led to higher OA values except at very high LEO ratios (Figure 1e and Figure 2e). The highest value of OA was obtained in the fiber-enriched beverage, using 27–65% LEO and lutein concentrations ≥1.75 mg/100 mL. The minimum acceptability score was attained for the fiber-free sample containing 1 mg/100 mL lutein and 100% ME in the mixture.

Other studies also reported the acceptable and improving effects of inulin and polydextrose [34,35], lutein [36], and ME [37] on the sensory properties of various functional/fortified foods and beverages. Few studies have been conducted on the acceptability of the addition of prebiotics to juices [38].

The physicochemical and sensory characteristics of a beverage result from individual components and physical and chemical interactions in the beverage matrix. Inulin is colorless and has a bland and neutral taste and aroma, without any off-flavor or aftertaste, and mixes easily with other ingredients without modifying their flavors. Its use offers the advantage of not compromising on taste while delivering nutritionally enhanced products [39]. Polydextrose is tasteless and has a low impact on flavor. Sometimes it helps to mask off-flavors that may come from some ingredients [40]. In addition, these two ingredients are multifunctional as sweetness enhancers, carbohydrate-based sugar, and fat replacers. Removing sugar from beverages decreases viscosity and thus reduces mouthfeel and body. Inulin and polydextrose can interact with other dissolved or dispersed molecular species in the hydrated state and provide different technological advantages, such as texturizing, thickening, emulsifying, stabilizing, or suspending. Therefore, their use can improve the mouthfeel of low-calorie beverages, and cover off-flavors in them [13,41].

**Figure 4:**
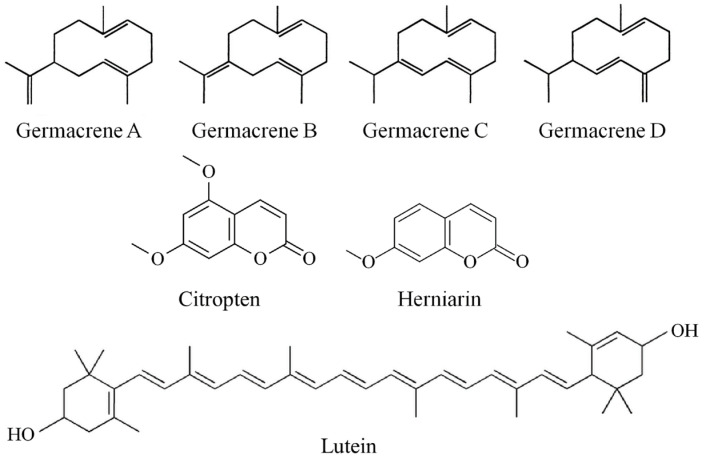
Structures of germacrene A, B, C, and D, citropten, herniarin, and lutein (black and white).

The orange-red color and sour taste of lutein (3,3′-dihydroxy-α-carotene) (Figure 4) caused a favorable change in the taste and appearance of the beverages and the LEO and ME imparted a savory strong taste and flavor to the beverages. Researchers reported that aromatic compounds in geraniol and vanillin were responsible for improving the quality characteristics of fiber-enriched strawberry juice [30]. There are detailed discussions of bioactive components and the volatile and key aroma-contributing molecules in citrus EOs, including LEO [42]. The LEO has a refreshing and sweetness-enhancing aroma. Besides the aldehydes and esters that are considered potent aroma contributors, germacrene A, B, C, and D (sesquiterpenes) (Figure 4), which are described as potent, warm, sweet, woody-spicy, geranium-like odor, are very important to the LEO aroma. Citropten (5,7-dimethoxycourmarin) and herniarin (7-methoxycourmarin) (Figure 4) are other compounds in LEO that have been described as sweet lactone-like and vanilla-like [9]. ME is known for its peculiar aroma and is a refreshing flavoring agent for foods and beverages. A wide spectrum of bioactive phytochemicals, such as flavonoids, phenolics, lignans, stilbenes, and EOs, are expected to be responsible for their aroma effects [43].

#### 3.1.3. Overall Optimization of the Variables

In the optimization, the point with the maximum desirability is selected. The desirability range is between 0 (completely undesirable response) and 1 (perfectly desirable response). For this purpose, the desired target for each response and factor was set to “within the range,” “minimum,” or “maximum,” and given each response′s importance and the study aim, a value of importance was selected for each response (Table S1). Finally, by applying the desirability function method, a combination of independent variables levels was obtained that had the maximum desirability. Six solutions for two combinations of categoric factor levels with desirability values corresponding to “very good desirability” (>80%) were suggested by the software. Optimization criteria, optimum points calculated using CD, and desirability values are shown in Table S1 and Table 4.

#### 3.1.4. Verification Experiments and Validation of the Model Equations

The experimental and predicted acceptance scores of sensory properties obtained at the optimum points and the error percentage between them are tabulated in Table 4. Only a small percentage error was observed between the experimental and predicted values and these values were reasonably close to each other. Thus, an acceptable percentage error (<30%) [44] indicated the validity and adequacy of the proposed response surface models and optimization method.

### 3.2. Determination of Physicochemical Properties

**Figure 5:**
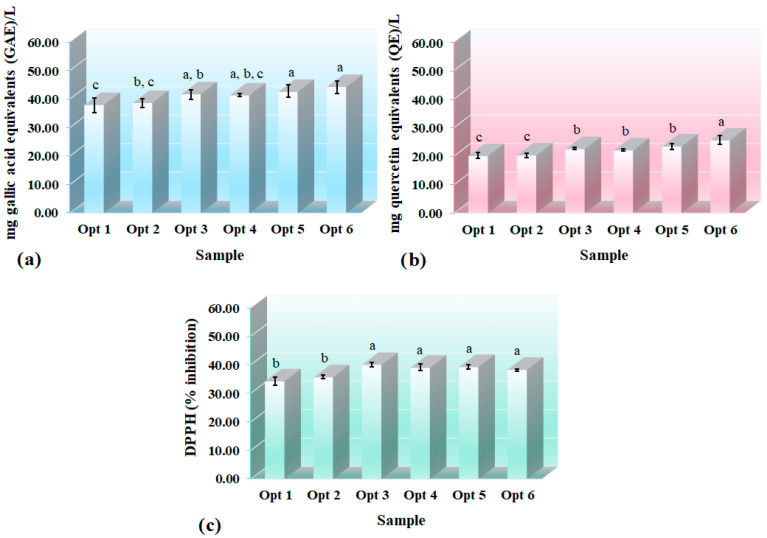
Bioactive compound content and in vitro antioxidant properties ((**a**) phenolic content, (**b**) flavonoid content, and (**c**) the DPPH^•^ scavenging activity) of the optimized formulations (Different letters indicate statistically significant differences (*p* ≤ 0.05)) (in color).

The data for the TPC and TFC of the six optimized formulations were in the range of 37.88–44.22 (mg GAE/L) and 20.04–25.49 (mg QE/L), respectively (Figure 5). All samples contained the highest amount of lutein used in beverage formulation. Increasing the ME proportion generally raised the TPC and TFC values of the beverages. This increment was expected given that ME is a rich source of these compounds. Sample Opt 6, which had the highest concentration of peppermint extract (ME) in the mixture, showed the maximum amounts of TPC and TFC (*p* < 0.05). The prebiotic fibers had an increasing effect on antioxidant capacity of the beverages (*p* < 0.05). On the other hand, the beverages containing intermediate levels of LEO and ME generally showed a non-significantly higher antioxidant capacity than the beverage containing a high proportion of ME. The sample Opt 3 containing prebiotic fibers, 3 mg/100 mL lutein and 48.24% LEO, and 51.76% ME, had the highest DPPH^•^ scavenging capacity (40.03 ± 0.80%), but this value was not significantly different from value obtained for sample Opt 6 (38.30 ± 0.39%) (*p* < 0.05). Thus, sample Opt 6 showed the highest TPC and TFC content and high antioxidant activity. According to the sensory evaluations, bioactive compounds content, and antioxidant capacity, sample Opt 6, which had prebiotics, 60.22% *v/v* ME, 39.78% *v/v* LEO in the mixture, and 3 mg/100 mL lutein, was chosen as the final optimal formulation. The TSS, pH, total acidity, and AA content of the optimized formulations are shown in Table S2. As it is expected, vitamin C degrades at 70 °C and this temperature is less than the one needed for the steam distillation process which is used for the extraction of herbal extracts. Therefore, the proportion of ME did not affect the vitamin C content of the beverages. Antioxidant properties of the developed functional beverage can be attributed to several used ingredients, including lime juice, lime peel essential oil, peppermint extract, lutein, and prebiotics.

Citrus (*Citrus* L. from Rutaceae) fruits, including *C. aurantiifolia*, are a rich source of nutrients and bioactive compounds, including AA and other vitamins, citric acid, essential minerals, and phenolic compounds (flavonoids and phenolic acids). Citrus flavonoids are particular nutrients in citrus because they are rare in other types of fruits. Flavanones are the major group of citrus phenolic compounds, among which hesperidin is the primary flavanone, followed by eriocitrin. Phenolic compounds are one of the significant contributors to antioxidant activity in citrus juice. Eriocitrin, which is stable even after the heat treatment process, has been found to have more potent antioxidant activity than other citrus flavonoids. AA is another crucial antioxidant and an efficient scavenger of reactive oxygen species (ROS) in citrus fruit juices. Unlike eriocitrin, it is thermolabile and is highly sensitive to light, as well as to various processing conditions [10,45,46]. There are several studies on the antioxidant potential of lutein [36] and prebiotics [47]. LEO is a complex mixture of organic compounds divided into three classes: terpenes (75%), oxygenated complexes (12%), and sesquiterpenes (3%). Limonene (monoterpene) has been reported to constitute the highest amount of volatile compounds. Additionally, there are approximately 20% of non-volatile chemicals in cold-pressed LEO. Besides colorants and wax, they are mainly coumarin and psoralene derivatives [9,46]. The DPPH scavenging activity of the LEO has been found to range from 10.65 to 66.44% in 0.08–3.46 mg/mL, with an IC50 value of 2.36 mg/mL [27]. The presence of terpenes, flavonoids, carotenes, and coumarins in citrus EOs is responsible for their strong antioxidative activities [17].

ME is rich in phenolic and flavonoid antioxidants and contains lower amounts of vitamins and terpenes. ME contains 0.75 g/L of different classes of polyphenolic compounds, mostly flavonoids (530 g/kg), phenolic acids (420 g/kg), lignans, and stilbenes (25 g/kg). The most abundant phenolics are eriocitrin, rosmarinic acid, eriodictyol-glycopyranosyl-rhamnopyranoside, and luteolin 7-O-rutinoside [15,48]. Antioxidant activity of *M. Piperita* extract and the direct positive correlation between its TPC and DPPH radical-scavenging have been reported [43,49,50]. The considerable variation in the results of the antioxidant potential of ME is due to the different antioxidant assay methods and ways of its expression. Phenolic acids (for example, caffeic acids and rosmarinic), flavones (for example, luteolin glycosides), and flavanones (for example, eriocitrin glycosides) are probably the major antioxidants, and vitamins (for instance carotenoids and ascorbic acid) are minor contributors to the overall antioxidant potential [51].

Our results revealed that the developed functional beverage is an acceptable source of bioaccessible health-related compounds. The antioxidant phytochemicals of the developed beverage (phenolic and terpenes, AA, prebiotics, lutein, etc.) have the potential for slowing or retarding and inhibiting the organic matter oxidation promoted by ROS and preventing biological structure damage and the development of oxidative stress- and inflammation-related diseases, such as diabetic and cardiovascular disorders, as well as some types of cancer [52]. Evidence suggests an inverse association between dietary fiber ingestion and inflammation and certain types of cancer, such as colon and breast cancer [12]. A wide variety of bioactivities has rendered inulin an outstanding natural nutrient [53]. Inulin is capable of scavenging ROS, which can help to alleviate oxidative stress, reduce lipid peroxidation in the stomach [47], and protect against hepatotoxicity [54]. Polydextrose is metabolized independently of inulin. It has health effects due to its laxative action and control of glucose and cholesterol levels in the blood. It helps to modulate appetite and satiety, causing a reduction in total caloric input and increasing antioxidant, antihypertensive, and antidiabetic activity [55,56].

## 4. Conclusions

New low-calorie fiber-enriched lime juice-based functional beverages containing prebiotics inulin/polydextrose, lutein, LEO, and ME were developed, and their sensory properties were optimized using the CD. The synergistic improving effects of these potential active components on the sensory properties resulted in beverages that were preferred by consumers. ANOVA showed a good fit of the developed regression equations to the data. Among the six optimized beverage formulations with “very good desirability” (>80%), the beverage with the highest amount of bioactive compounds content and antioxidant capacity was selected as the last optimal beverage. This beverage was the one with inulin/polydextrose at 20% DRV, 3 mg/100 mL lutein, and a 1.99:3.01 (mL: mL) LEO: ME mixture combination, which contained 44.22 mg GAE/L of TPC, 25.49 mg QE/L of TFC, and exhibited 38.30% DPPH^•^ scavenging activity. The validity and adequacy of the proposed models were verified experimentally. The newly designed beverage with good organoleptic properties has the potential to promote health for people with diabetes and hypertension and meets the consumer demand for nutritious and healthy beverages; therefore, it has good potential for commercialization.

## Figures and Tables

**Table 1:** Design of experiment for the optimization of the functional beverage formulation and corresponding sensory attributes, levels of independent variables, and values of the responses.

Design Point.	Components		Factors		Responses g									
	Mixture Components		Numeric Factor	Categoric Factor	Taste		Flavor		Texture		Color		Overall Acceptance	
	*X*_1_: LEO Solution (0.1 *v/v*) % ^a^ (mL) ^c^	*X*_2_: ME % ^b^ (mL) ^c^	*X*_3_: Lu (mg/100 mL)	*X*_4_: I/P Mixture ^d^	Exp. V. ^e^	Pre. V. ^f^	Exp. V.	Pre. V.	Exp. V.	Pre. V.	Exp. V.	Pre. V.	Exp. V.	Pre. V.
1	50.00 (2.50)	50.00 (2.50)	3.00	Level 1	7.3 ± 1.5	7.2	7.9 ± 1.4	7.9	7.2 ± 1.4	7.2	7.1 ± 1.7	7.0	8.3 ± 1.2	7.9
2	50.00 (2.50)	50.00 (2.50)	3.00	Level 2	7.6 ± 1.2	7.8	7.7 ± 1.6	7.9	7.2 ± 0.7	6.7	6.6 ± 1.4	6.8	8.3 ± 1.2	8.2
3	0.00 (0.00)	100.00 (5.00)	3.00	Level 1	5.3 ± 1.6	5.3	6.4 ± 0.9	6.3	6.5 ± 2.1	6.4	4.3 ± 1.2	4.3	6.3 ± 1.7	6.3
4	100.00 (5.00)	0.00 (0.00)	3.00	Level 1	6.0 ± 1.5	6.0	6.9 ± 1.0	6.8	8.1 ± 1.7	8.0	7.1 ± 1.7	6.9	6.6 ± 1.1	6.6
5	75.00 (3.75)	25.00 (1.25)	2.00	Level 1	6.5 ± 0.9	6.8	7.6 ± 1.2	7.7	7.7 ± 1.2	7.5	6.8 ± 1.4	6.6	7.0 ± 0.9	7.4
6	100.00 (5.00)	0.00 (0.00)	1.00	Level 2	6.0 ± 0.7	6.0	6.8 ± 1.3	6.8	6.0 ± 1.6	5.9	5.9 ± 1.1	5.8	5.9 ± 0.9	6.0
7	0.00 (0.00)	100.00 (5.00)	1.00	Level 1	5.3 ± 0.8	5.2	6.5 ± 1.7	6.3	6.2 ± 1.3	6.2	4.5 ± 1.6	4.4	5.3 ± 1.2	5.3
8	50.00 (2.50)	50.00 (2.50)	3.00	Level 1	7.2 ± 1.1	7.2	7.8 ± 1.8	7.9	7.0 ± 1.0	7.2	6.9 ± 2.0	7.0	7.7 ± 1.3	7.9
9	50.00 (2.50)	50.00 (2.50)	1.00	Level 1	7.0 ± 1.3	6.9	7.9 ± 1.5	7.9	7.1 ± 1.3	7.1	5.8 ± 0.8	5.7	7.6 ± 1.2	7.6
10	50.00 (2.50)	50.00 (2.50)	1.00	Level 1	6.9 ± 1.9	6.9	7.8 ± 1.4	7.9	6.9 ± 2.3	7.1	5.6 ± 1.2	5.7	7.7 ± 1.7	7.6
11	100.00 (5.00)	0.00 (0.00)	3.00	Level 2	6.3 ± 1.1	6.3	6.6 ± 2.1	6.8	5.9 ± 1.8	6.0	6.8 ± 1.4	7.1	6.5 ± 1.6	6.5
12	0.00 (0.00)	100.00 (5.00)	1.00	Level 2	5.6 ± 1.5	5.5	6.1 ±1.8	6.3	7.5 ± 1.9	7.3	4.5 ± 1.7	4.4	6.3 ± 1.8	6.1
13	100.00 (5.00)	0.00 (0.00)	1.00	Level 1	5.8 ± 1.2	5.7	7.0 ± 1.5	6.8	8.0 ± 1.7	7.9	5.4 ± 1.2	5.6	6.2 ± 1.0	6.1
14	25.00 (1.25)	75.00 (3.75)	2.00	Level 2	7.2 ± 1.6	7.0	7.9 ± 1.4	7.4	6.4 ± 0.7	7.0	5.7 ± 1.5	5.5	7.7 ± 1.3	7.7
15	100.00 (5.00)	0.00 (0.00)	2.00	Level 2	6.3 ± 1.2	6.2	6.8 ± 1.9	6.8	5.9 ± 1.7	5.9	6.7 ± 1.3	6.4	6.3 ± 0.9	6.2
16	0.00 (0.00)	100.00 (5.00)	2.00	Level 1	5.3 ± 1.6	5.3	6.5 ± 0.8	6.3	6.3 ± 0.7	6.3	4.4 ± 1.3	4.4	5.8 ± 1.2	5.8
17	0.00 (0.00)	100.00 (5.00)	1.00	Level 2	5.4 ± 1.3	5.5	5.9 ± 0.5	6.3	7.3 ± 0.7	7.3	4.3 ± 1.3	4.4	6.0 ± 1.1	6.1

^a^ This value represents the concentration distribution of the LEO concerning the total LEO + ME amount. ^b^ This value represents the concentration distribution of the ME concerning the total LEO+ME amount. ^c^ Amount added to 100 mL of beverage formulation. ^d^ The levels of categoric factor indicate either the absence (Level 1) or presence (Level 2) of I/P. ^e^ Experimental value. ^f^ Predicted value. ^g^ Values are presented as mean ± SD, n = 3.

**Table 2:** Combined model mixture process fit summary and analysis of variance (partial sum of squares).

Source	Suggested Models		Sequential *p*-Value		Partial Sum of Squares		Lack of Fit (LOF)	Model Summary Statistics (MSS)		
	Mix Order	Process Order	Mix	Process	Sum of Squares	Mean Square		R^2^	Adj-R^2^	Pred-R^2^
Taste	Quadratic	Linear	<0.001 **	0.011 *	10.04	1.26	0.27	0.98	0.96	0.92
Flavor	Quadratic	Mean	<0.001 **	-	7.03	3.52	0.11	0.91	0.90	0.87
Texture	Linear	Linear	<0.001 **	<0.001 **	7.07	1.41	0.13	0.90	0.86	0.80
Color	Quadratic	Linear	0.001 **	0.003 **	17.71	2.21	0.12	0.97	0.95	0.84
Overall acceptance	Quadratic	Linear	<0.001 **	0.033 *	12.90	1.61	0.64	0.97	0.94	0.90

*^,^ ** Significant at *p*-level ˂ 0.05 and *p*-level ˂ 0.01, respectively.

**Table 3:** Regression coefficients for the response variables and analysis of variance of the regression models.

Source	Taste			Flavor			Texture			Color			Overall Acceptance		
	Reg. Co. ^a^	F-Value	*p*-Value	Reg. Co.	F-Value	*p*-Value	Reg. Co.	F-Value	*p-*Value	Reg. Co.	F-Value	*p*-Value	Reg. Co.	F-Value	*p*-Value
X_1_	5.99	-	-	6.80	-	-	6.95	-	-	6.35	-	-	6.27	-	-
X_2_	5.42	-	-	6.29	-	-	6.83	-	-	4.39	-	-	6.21	-	-
X_1 × 2_	6.46	235.68	<0.001 **	5.28	125.28	<0.001 **	-	-	-	3.59	31.63	0.001 **	6.58	120.76	<0.001 **
X_1 × 3_	0.14	2.92	0.126	-	-	-	0.05	0.19	0.672	0.65	27.87	0.001 **	0.25	4.96	0.057
X_1 × 4_	0.17	5.44	0.048 *	-	-	-	−1.01	93.16	<0.001 **	0.09	0.66	0.440	−0.04	0.15	0.712
X_2 × 3_	0.05	0.21	0.660	-	-	-	0.10	0.53	0.482	−0.04	0.05	0.835	0.49	9.57	0.015 *
X_2 × 4_	0.15	2.66	0.142	-	-	-	0.53	20.78	0.001 **	0.01	0.01	0.941	0.40	9.56	0.015 *
X_1 × 2 × 3_	0.18	0.20	0.670	-	-	-	-	-	-	1.30	4.47	0.067	−0.79	1.87	0.209
X_1 × 2 × 4_	0.57	1.82	0.214	-	-	-	-	-	-	−0.61	0.91	0.369	−0.10	0.50	0.500
Model	-	48.42	<0.001 **	-	69.42	<0.001 **	-	20.13	<0.001 **	-	37.08	0.001 **	-	30.77	<0.001 **
Linear mixture	-	36.22	0.001 **	-	13.55	0.003 **	-	0.46	0.513	-	172.68	<0.001 **	-	3.98	0.081
LOF ^b^	-	2.21	0.273	-	4.88	0.109	-	4.26	0.130	-	4.63	0.119	-	0.75	0.636
R^2^	0.98	-	-	0.91	-	-	0.90	-	-	0.97	-	-	0.97	-	-
R^2^_adj_^c^	0.96	-	-	0.90	-	-	0.86	-	-	0.95	-	-	0.94	-	-
R^2^_pred_^d^	0.92	-	-	0.87	-	-	0.80	-	-	0.84	-	-	0.90	-	-
Adeq. Precision ^e^	21.66	-	-	16.67	-	-	13.52	-	-	15.44	-	-	17.36	-	-
C.V. ^f^ %	2.57	-	-	3.19	-	-	3.85	-	-	4.23	-	-	3.38	-	-
Std. Dev. ^g^	0.16	-	-	0.23	-	-	0.26	-	-	0.24	-	-	0.23	-	-
PRESS	0.85	-	-	1.02	-	-	1.53	-	-	2.91	-	-	1.29	-	-

*^,^ ** Significant at *p*-level ˂ 0.05 and *p*-level ˂ 0.01, respectively. ^a^ Regression coefficient. ^b^ Lack of fit. ^c^ Adjusted R^2^. ^d^ Predicted R^2^. ^e^ Adequate precision. ^f^ Coefficient of variation. ^g^ Standard deviation.

**Table 4:** Optimum points calculated using CD, desirability values, the actual and theoretical acceptance scores of responses obtained at the optimum points, and the percentage errors between these values.

Optimized Sample	Optimum Formula				Desirability	Responses at Optimum Point					
	Mixture Components		Numeric Factor	Categoric Factor							
	LEO Solution (0.1 *v/v*) (%)	ME (%)	Lutein (mg/100 mL)	I/P Mixture			Taste	Odor	Texture	Color	Overall Acceptance
						TV ^a^	7.17	7.87	7.36	7.20	7.88
Opt 1	59.71	40.29	3.00	Level 1	0.87	AV ^b^	6.7 ± 1.4	6.9 ± 1.3	6.9 ± 1.1	7.4 ± 1.5	7.1 ± 1.6
						PE ^c^ (%)	−7.3	−14.6	−6.7	3.2	−10.4
						TV	7.18	7.87	7.34	7.17	7.89
Opt 2	58.01	41.99	3.00	Level 1	0.87	AV	6.5 ± 1.7	7.0 ± 1.4	7.6 ± 1.2	6.9 ± 1.1	6.9 ± 1.7
						PE (%)	−10.4	−12.8	2.8	−4.4	−13.8
						TV	7.75	7.86	6.75	6.75	8.22
Opt 3	48.24	51.76	3.00	Level 2	0.82	AV	6.5 ± 1.2	6.8 ± 1.6	7.1 ± 1.1	6.0 ± 1.5	6.6 ± 1.2
						PE (%)	−18.6	−15.3	4.3	−12.4	−24.0
						TV	7.76	7.87	6.72	6.79	8.21
Opt 4	50.00	50.00	3.00	Level 2	0.82	AV	6.9 ± 1.0	7.4 ± 1.4	6.3 ± 1.2	5.8 ± 1.6	7.2 ± 1.2
						PE (%)	−13.3	−6.8	−6.1	−17.8	−14.1
						TV	7.72	7.84	6.78	6.68	8.23
Opt 5	45.89	54.11	3.00	Level 2	0.82	AV	7.9 ± 0.7	7.5 ± 1.2	7.5 ± 1.3	7.0 ± 1.2	8.2 ± 0.6
						PE (%)	2.0	−4.7	9.6	4.9	−0.2
Opt 6	39.78	60.22	3.00	Level 2	0.81	TV	7.62	7.76	6.87	6.47	8.22
						AV	6.5 ± 1.4	7.1 ± 1.3	7.5 ± 1.1	6.7 ± 1.5	6.9 ± 1.5
						PE (%)	−16.9	−10.1	8.4	3.2	−19.1

Data are presented as mean ± SD, n = 3. ^a^ Theoretical value. ^b^ Actual value (Mean ± S.D). ^c^ Percentage error.

## Data Availability

The data presented in this study are available on request from the corresponding author.

## Supplementary Materials

The following supporting information can be downloaded at: https://www.mdpi.com/article/10.3390/foods12030680/s1, Figure S1: Protocol for the preparation of 1 L functional beverages in a laboratory scale; Table S1: Optimization criteria and dependent variables optimization; Table S2: Physicochemical properties of the optimized formulations. References [57,58] cited in Supplementary Materials.
