# Supplementary material for: Development of a Novel Low-Calorie Lime Juice-Based Prebiotic Beverage Using a Combined Design Optimization Methodology
Source: Foods. 2023 Feb 3;12(3):680. doi: 10.3390/foods12030680 (PMC9914248; doi:10.3390/foods12030680)
Supplement: Supplementary file 1 [file foods-12-00680-s001.zip › foods-2173302-supplementary.pdf]

Supporting Information

Development of a Novel Low-Calorie Lime Juice-Based Prebiotic Beverage Using a Combined Design Optimization Methodology

Leila Abolghasemi Fakhri [1], Babak Ghanbarzadeh [1,*] and Pasquale M. Falcone [2,*]

[1] Department of Food Science and Technology, Faculty of Agriculture, University of Tabriz, Tabriz P.O. Box 51666-16471, Iran

[2] Food and Environmental Sciences, University Polytechnical of Marche—Department of Agricultural, Brecce Bianche 10, 60131 Ancona, Italy

* Correspondence: authors: ghanbarzadeh@tabrizu.ac.ir (B.G.); pm.falcone@univpm.it (P.M.F.)

Material and methods

Preparation of iodine solution

To prepare of the iodine solution, 5 g potassium iodide (KI) and 0.268 g potassium iodate (KIO_3) were dissolved in 200 ml of distilled water. Then, 30 ml of 3 M sulfuric acid (H_2SO_4) was added and diluted with distilled water to the 500 ml solution.

Titratable acidity (TA)

The TA was determined by titrating a 10 mL sample with NaOH (0.1 N) using phenolphthalein till the sample turned light pink (pH 8.1) [1] and was calculated as [2]:

$$\%TA = \frac{V \times 0.1N\ NaOH \times 0.064 \times 100}{V_s}$$

Where V is the titer volume of NaOH (ml) and V_s is the volume of the sample (ml). Results were expressed as % citric acid in TA and ºBrix in TSS.

Table S1. Optimization criteria and dependent variables optimization

Variables/Responses	Goal	Lower limits	Upper limits	Weights	Importance
**LEO solution (0.1 v/v) (%)**	In range	0	100	1	3
**ME (%)**	In range	0	100	1	3
**Lutein (mg/100 ml)**	In range	1	3	1	3
**Prebiotics**	In range	Level 1	Level 2	1	3
**Taste**	Maximize	5.25	7.62	1	5
**Flavor**	Maximize	5.85	7.92	1	5
**Texture**	Maximize	5.90	8.05	1	4
**Color**	Maximize	4.30	7.12	1	5
**Overall acceptance**	Maximize	5.28	8.25	1	5

Table S2. Physicochemical properties of the optimized formulations

Optimized sample	TSS (ºBrix)	pH	Ascorbic acid[a]	Titratable acidity[b]
**Opt 1**	1.0 ± 0.0	2.62 ± 0.07[a]	23.28 ± 1.54[a]	0.56 ± 0.01[c]
**Opt 2**	1.0 ± 0.0	2.59 ± 0.03[a]	22.33 ± 1.27[a]	0.56 ± 0.04[c]
**Opt 3**	3.5 ± 0.0	2.48 ± 0.02[b]	25.16 ± 0.64[a]	0.60 ± 0.02[b, c]
**Opt 4**	3.5 ± 0.0	2.46 ± 0.04[b]	23.46 ± 1.65[a]	0.64 ± 0.04[a, b]
**Opt 5**	3.5 ± 0.0	2.41 ± 0.04[b]	25.11 ± 1.92[a]	0.65 ± 0.03[a]
**Opt 6**	3.5 ± 0.0	2.33 ± 0.03[c]	24.43 ± 1.52[a]	0.68 ± 0.01[a]

Data are presented as mean ± SD, n = 3.

[a] mg/100 g

[b] g of citric acid/100 ml

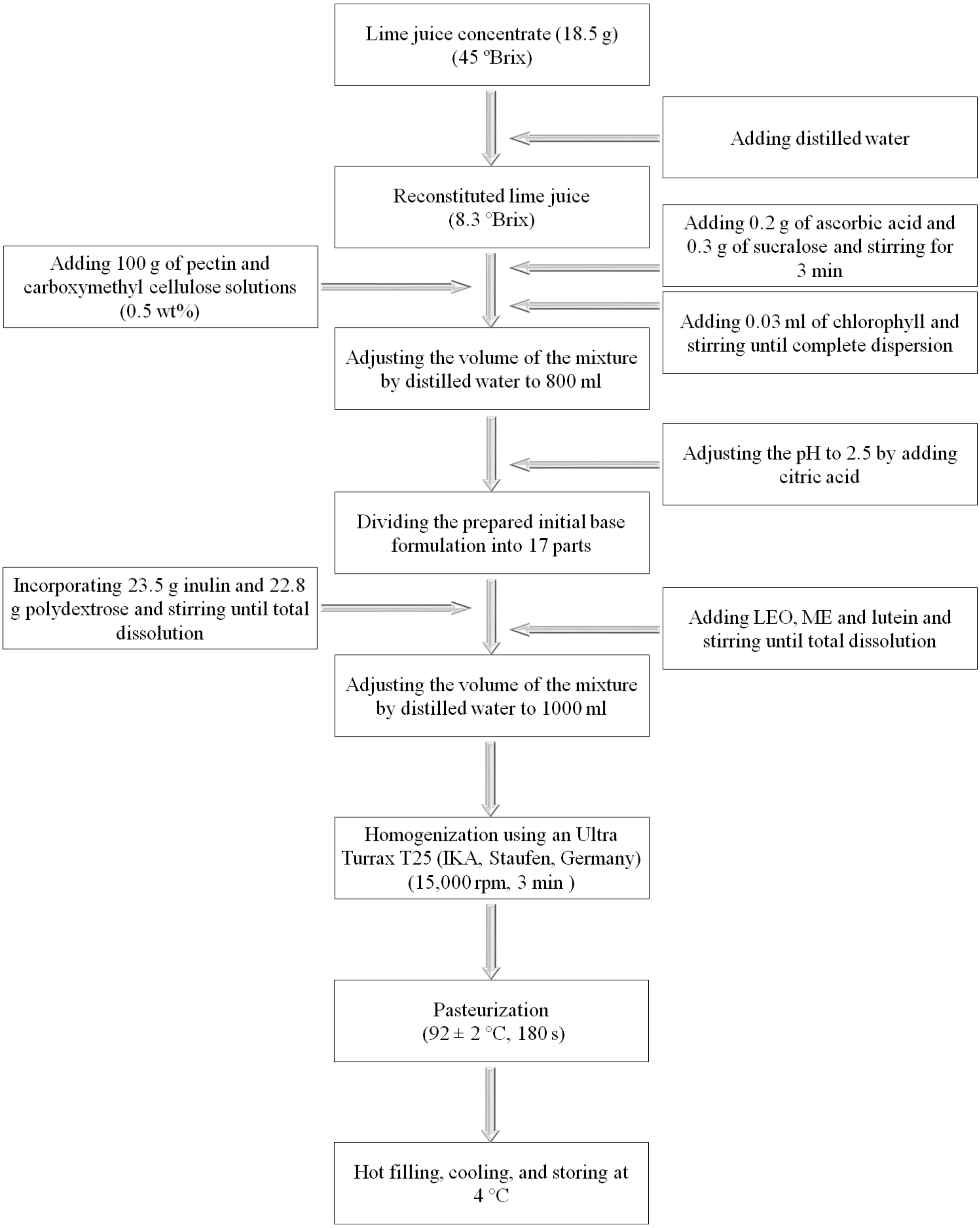

Figure S1. Protocol for the preparation of 1 L functional beverages in a laboratory scale. (black and white)

References

1. AOAC. AOAC official method 942.15: Acidity (titratable) of fruit products. *Official Methods of Analysis of AOAC International* **1996**.
2. Tyl, C.; Sadler, G.D. pH and titratable acidity. In *Food analysis*, Springer: 2017; pp. 389-406.
